# Association between maternal glucose levels during pregnancy and gestational diabetes mellitus: an analytical cross-sectional study

**DOI:** 10.1186/s13098-015-0013-8

**Published:** 2015-03-12

**Authors:** Gisele Seabra, Cláudia Saunders, Patrícia de Carvalho Padilha, Lenita Zajdenverg, Letícia Barbosa Gabriel da Silva, Marta Maria Antonieta de Souza Santos

**Affiliations:** Universidade Federal do Rio de Janeiro (UFRJ) – Centro de Ciências da Saúde, Instituto de Nutrição Josué de Castro, Cidade Universitária, Av. Carlos Chagas Filho, 373, bl. J, 2°. andar, Rio de Janeiro, CEP. 21941.590 RJ Brasil; Maternidade Escola (UFRJ), Rio de Janeiro, RJ Brasil; Serviço de Nutrologia/Departamento de Clínica Médica e Maternidade Escola, Rio de Janeiro, Brasil

**Keywords:** Fasting glucose, Gestational diabetes mellitus, Complications

## Abstract

**Background:**

To evaluate the association between fasting glucose levels in women throughout pregnancy and the occurrence of gestational diabetes mellitus (GDM) and other pregnancy complications, macrosomia, and cesarean delivery.

**Methods:**

An analytical cross-sectional study with 829 healthy pregnant women receiving health care at a public maternity unit in Rio de Janeiro between 1999 and 2008. The dependent variables assessed in the study were: GDM (was confirmed when two or more values were above the glucose curve using 100 g glucose), complications, mode of delivery and birth weight. Macrosomia was defined as a birth weight of >4000 g. The independent variables assessed were: maternal fasting glucose per trimester as a continuous variable, divided into three categories, socio-demographic data on the mothers. The level of statistical significance was set at 5%.

**Results:**

The mean fasting glucose levels of the women who had GDM were higher in the second trimester than for those who had no pregnancy complications (90.5 mg/dL vs. 78.5 mg/dL, p = 0.000). Higher mean fasting glucose levels were also found in the third trimester for women who developed GDM than for those with no pregnancy complications (90 mg/dL vs. 77.8 mg/dL, p = 0.016). Women who had a cesarean delivery had higher fasting glucose levels in the second (80.4 mg/dL vs. 78 mg/dL, post hoc = 0.034) and third (80.4 mg/dL and 77.1 mg/dL; post hoc = 0.005) trimesters than women who had a normal delivery. Also, higher fasting glucose levels were found in the second semester for women whose infants had macrosomia than for women whose newborns were normal weight (86.2 mg/dL and 78.8 mg/dL; post hoc = 0.003). The chance of develop GDM was higher for the women with glucose levels in the 90–94 mg/dL range in the second trimester (OR = 7.2; 95% CI = 2.33-22.24) than for the women whose glucose levels were in the <80 mg/dL and 80-90 mg/dL ranges.

**Conclusion:**

Second and third trimester fasting glucose levels below the cut-off values for the diagnosis of GDM are associated with an increased risk of pregnancy complications. The dependent variables assessed in the study were: GDM (present/absent), complications, mode of delivery (normal, forceps, cesarean), and birth weight.

## Introduction

There is a consensus in the literature that pregnant women with gestational diabetes mellitus (GDM) are more likely to have adverse perinatal outcomes, such as fetal macrosomia, the need for a surgical delivery, and birth injuries. Furthermore, such women are more likely to develop diabetes mellitus in the future, which is seen in between 20% and 50% of such cases up to ten years after childbirth [1-3]. However, this risk is disputed when maternal glucose is found to be high, but not high enough to be diagnosed as GDM [4,5].

GDM is defined as any degree of glucose intolerance with onset or first recognition during pregnancy [2]. The prevalence of GDM in adult women (>20 years of age) receiving health care from the Brazilian public health system, according to the previous diagnostic criteria set forth by the World Health Organization (WHO), was 7.6% in the 1980 s [6,7].

The Hyperglycemia and Adverse Pregnancy Outcomes [4] (HAPO) multicenter study was pioneering for demonstrating an association between moderately higher levels of maternal glucose and perinatal complications. A significant and rising likelihood of fetal macrosomia, the need for delivery by caesarean section, umbilical cord C-peptide, and neonatal hypoglycemia, was found to be associated with increased maternal glucose levels [4].

The following year, Riskin-Mashiah *et al.* [5] also published a study that demonstrated an association between fasting glucose levels in the first trimester and the progressively increased occurrence of pregnancy complications and adverse outcomes: GDM, fetal macrosomia, and the need for delivery by cesarean section.

In a retrospective cross-sectional study, a significant association was found between GDM risk factors or elevated glucose levels at the first antenatal visit and the occurrence of the following outcomes: large for gestational age, increased indication of cesarean delivery, and preterm birth [8].

In the light of new evidence that shows there is an association between maternal glucose concentrations below the cut-off points for the diagnosis of GDM and adverse outcomes, a new proposal for the diagnosis of GDM was recommended in 2010 by the International Association of Diabetes and Pregnancy Study Groups (IADPSG) [9] and endorsed by the WHO [2].

The most common complications affecting maternal and fetal health associated with GDM are fetal macrosomia and the need for a surgical delivery. Macrosomia increases the chance of complications like systemic hypertension, preterm birth, infections, intrauterine death, shoulder dystocia, fractured clavicle and humerus, and other birth injuries [10,11]. Meanwhile, cesarean deliveries have been associated with complications such as the increased chance of infections, the need for blood transfusions, pneumonia, the increased risk of respiratory distress in the newborn [12], and a three times higher chance of breastfeeding being terminated after 30 days of life because of the need for artificial incubation [13].

The aim of this study was to analyze the association between maternal glucose levels during pregnancy and the development of pregnancy complications (e.g. anemia, pregnancy-induced hypertension [PIH], urinary tract infections, premature amniorrhexis, GDM), fetal macrosomia, and surgical delivery (forceps or cesarean) in adult women who screened negative for diabetes mellitus.

## Methods and study design

This analytical cross-sectional study [14] is based on data from a study at a public maternity unit in Rio de Janeiro/Brazil. The unit provides outpatient antenatal services for low- and high-risk pregnancies, and free care for pregnant adults and adolescents who live in the state of Rio de Janeiro. The antenatal care is provided by a multi-disciplinary team that includes obstetricians, nutritionists, nutrologists, nurses, psychologists, psychiatrists, social workers and other professionals.

The population under study was made up of adult pregnant women and recent mothers who received health care between 1999 and 2008. Specifically, they were women and their respective newborns who received health care at the maternity unit in question at three different periods: 225 from April 1999 to December 2001; 227 from June 2005 to September 2006, and 394 from September 2007 to September 2008. Of this original total of 846 women, 829 met the inclusion criteria, namely: having received antenatal care and/or given birth at the maternity unit under study; aged ≥20; singleton pregnancy; no chronic diseases prior to pregnancy; plasma glucose screened and recorded throughout pregnancy.

The number of pregnant women accompanied by trimester of pregnancy was 317 in the first trimester, 288 and 224 in the second and third trimesters respectively.

Maternal plasma glucose was measured using the glucose oxidase method, and cases of GDM were identified using the criteria valid during the period in question.

When a glucose level of ≥ 130 mg/dL was obtained 1 hour after the oral glucose tolerance test (OGTT) with 50 g (screening test), the pregnant women were referred to conduct another OGTT with 100 g anhydrous glucose 1 h, 2 h and 3 h after ingestion. GDM were confirmed when two or more values were greater than or equal to 95 mg/dL (fasting), 180 mg/dL (1 h), 155 mg/dL (2 h), 140 mg/dL (3 h) [15,16].

As well as fasting plasma glucose, other maternal characteristics studied included GDM, anemia, PIH, urinary tract infection, and premature amniorrhexis.

The mode of delivery was also investigated, as were socio-demographic and obstetric characteristics, anthropometric data, and details on the antenatal care received (number of prenatal medical visits, and number of antenatal visits with a nutritionist).

The neonatal conditions evaluated were weight at birth, with underweight being <2500 g, normal weight being 2500 g to 3990 g, and overweight being ≥4000 g, irrespective of gestational age [17].

The maternal characteristics, information about antenatal care, pregnancy complications, and neonate characteristics were identified by consulting the opinions issued by the antenatal team and/or by interpreting the results of the exams in the women’s medical records, which was done by a trained, supervised team.

The dependent variables assessed in the study were: GDM (present/absent), complications, mode of delivery (normal, forceps, cesarean), and birth weight.

The independent variables assessed were: maternal fasting glucose per trimester as a continuous variable, divided into three categories: <80 mg/dL, 80-90 mg/dL and 90- 94 mg/dL; socio-demographic data on the mothers (age, education, marital status, basic sanitation conditions, total household income); anthropometric data (pre-pregnancy BMI and weight, weight gain per trimester, total weight gain); and antenatal care. The basic sanitation conditions were considered adequate when the women’s homes had piped, treated water, sewerage, and regular waste collection; they were deemed inadequate in the absence of any of these services.

Pregnancy complications were identified from the medical records and by interpreting exams. Anemia was diagnosed when hemoglobin levels were <11 g/dL at any time during pregnancy [18]. PIH was identified when blood pressure after the twentieth week of pregnancy was ≥140 × 90 mmHg. Women diagnosed as having hypertension were also investigated for the presence of proteinuria, with >300 mg protein in urine in 24 h being interpreted as indicative of preeclampsia, and this value in association with seizures as being eclampsia. Other complications, like urinary infection and premature rupture of membranes, were identified by consulting the medical records [18,19].

### Statistical analyses

To evaluate the quality of the data, the concordance of the responses obtained by two different researchers was measured, based on the information they collected from the same medical records from the cohorts from 1999 (n = 35) and 2005 (n = 28) [20]. The statistics used for this analysis were intraclass correlation coefficient (ICC) and kappa (k), with ICC and k > 0.61 being taken to indicate good concordance [20].

An exploratory data analysis was conducted, the means and standard deviation (SD) were calculated, Student’s t-test, ANOVA and post-hoc Tukey test were performed, and Levene’s test was used to test homogeneity of variance*.* The level of statistical significance was set at 5%. Women who developed GDM in the second trimester were excluded from the analyses of the mean fasting glucose levels in the third trimester of pregnancy.

Bivariate analysis was used to evaluate the association between the dependent and independent variables using the chi-squared test, considering p < 0.05. Next, logistic regression models were tested to identify the predictors of the dependent variables (adverse outcomes – GDM, cesarean delivery, and fetal macrosomia). Logistic regression was used to estimate the odds ratios for the outcomes evaluated with a confidence interval (CI) of 95%. The forward stepwise method was used to adjust the multivariate model, with p-values of 0.05 and 0.10 for entry and exit, respectively, to test all the independent variables. Only those variables with p < 0.05 were kept in the final model. All the analyses were conducted using *SPSS for Windows*, version 17.0.

The original study that yielded the database for this article was designed in compliance with resolution 196/96 of the National Health Council (Conselho Nacional de Saúde; Brasil, 1996). It was approved by the research ethics committee of the National School for Public Health, Fundação Oswaldo Cruz (opinion #75/02), and by the ethics committee of Instituto de Puericultura e Pediatria Martagão Gesteira at the Federal University of Rio de Janeiro (UFRJ) on December 14, 2004 (opinion # 35/04) and the research ethics committee of the maternity unit, Maternidade Escola da UFRJ on October 8, 2008 (CEP – 0013.0.361.000-08).

## Results

In this study, three cohorts of pregnant women who received care in different time periods at the maternity unit in question were studied. As over the periods studied, some changes took place in the number of visits to the nutritionist during antenatal care, the impact of nutritional supervision (yes/no) on the outcomes of interest in this study was evaluated, namely: mode of delivery (normal/cesarean; p = 0.172); weight at birth (underweight and normal weight/macrosomia; p = 0.807); neonatal complications (yes/no; p = 0.357); pregnancy complications (yes/no; p = 0.919). The differences between mean maternal glucose levels in the second trimester (with nutritional supervision – 79.79 mg/dL; without nutritional supervision – 78.99 mg/dL; p = 0.234) and the third trimester (with nutritional supervision – 79.56 mg/dL; without nutritional supervision – 77.91 mg/dL; p = 0.121) were also investigated. In view of the results obtained, the data analysis was done on the sum of women from the cohorts in this study, because no differences were encountered between them.

In the data quality evaluation, good inter-rater concordance indices were found, and it was also found that the procedures for obtaining the information were the same for both groups. The values calculated were ICC >0.92 and k >0.65 [20].

The average age of the women was 27.6 (SD = 5.2); 35.4% had white skin, 97.2% lived in housing with adequate basic sanitation, and 32.4% had completed 12 years of schooling. In the anthropometric evaluation, the average pre-pregnancy BMI was found to be 23.7 kg/m^2^ (n = 782; SD = 8.1). Most of the women (63.8%) were classified as normal weight at the start of pregnancy, and 38.4% had adequate total weight gain during pregnancy, according to Institute of Medicine criteria (Table 1) [17].

**Table 1:** **Characteristics of the women and their newborns (Rio de Janeiro, Brazil - 1999 and 2008)**

***Characteristic***	***N***	***%***
**Pre-pregnancy nutritional status (BMI)**		
Underweight (<18.5 kg/m^2^)	84	10.9
Normal weight (18.5 – 24.99 kg/m^2^)	494	63.8
Overweight (25.0 – 29.99 kg/m^2^)	131	16.9
Obese (≥30.0 kg/m^2^)	65	8.4
**Total weight gain**		
Low	209	26.9
Intermediate	298	38.4
High	270	34.7
**Mode of delivery**		
Normal	288	49.6
Forceps	22	3.8
Cesarean section	270	46.6
**Gestational age at birth**		
Preterm (<37 weeks)	26	4.7
Term (≥37 weeks)	527	95.3
**Weight at birth**		
Low (<2500 g)	33	4.3
Normal (2500 g - 3999 g)	695	91.1
High (≥4000 g)	35	4.6
**Pregnancy complications**		
None	455	54.9
Other	347	41.9
Gestational diabetes mellitus	27	3.3

The mean fasting glucose levels in the first, second and third trimesters were 79.1 mg/dL (n = 337; SD = 10.2), 79.3 mg/dL (n = 499; SD = 10.8) and 78.8 mg/dL (n = 414; SD = 10.5), respectively.

The women had an average of 8.2 antenatal consultations with an obstetrician (n = 825; SD = 2.3), and 1.7 antenatal consultations with a nutritionist (n = 827; SD = 2.1). The average gestational age at birth was 39.1 weeks (n = 743; SD = 1.6) and the average weight of the newborns was 3.2 kg (n = 764; SD = 4.8).

Table 1 shows the characteristics of the pregnancies, deliveries and postpartum periods. It shows that 54.9% of the women had no pregnancy complications, and 49.6% had a vaginal delivery; 95.3% of the infants were term births, and 4.6% had macrosomia. Further, it was found that 3.3% (n = 27, Table 1) of the women studied developed GDM. The other pregnancy complications observed were anemia (n = 191; 23%), PIH (n = 40; 4.8%), premature amniorrhexis (n = 65; 7.8%), and urinary tract infection (n = 23; 2.8%).

A significant association was found between maternal glucose levels in the second (p = 0.000) and third (p = 0.016) trimesters and the development of GDM (Table 2). The mean fasting glucose levels were higher amongst the group who developed GDM (second trimester: 90.5 mg/dL; third trimester: 90 mg/dL) than amongst women who did not have any complications during pregnancy (second trimester: 78.5 mg/dL; third trimester: 77.8 mg/dL).

**Table 2:** **Maternal fasting plasma glucose levels per trimester and pregnancy complication (Rio de Janeiro, Brazil - 1999 and 2008)**

***Complication***	***Fasting plasma glucose in the first trimester (mg/dL)***				***Fasting plasma glucose in the second trimester (mg/dL)***				***Fasting plasma glucose in the third trimester (mg/dL)*** ^***a***^			
	**n**	**Mean**	**SD**	**p**	**n**	**mean**	**SD**	**p**	**n**	**mean**	**SD**	**p**
None	196	79.3	9.3	0.479	270	78.5	9.8	0.000	220	77.8	9.6	0.016
GDM	1	86	-		18	90.5	21.3		4	90	19.4	

^*a*^Fasting plasma glucose in the third trimester.

As Table 3 shows, a significant association was found between maternal fasting plasma glucose concentrations in the second trimester and two outcomes: weight at birth (p = 0.005) and mode of delivery (p = 0.039). Significantly higher mean glucose levels were found for the mothers of the infants with macrosomia than for the mothers of the normal weight infants (86.2 mg/dL and 78.8 mg/dL, respectively, p = 0.003). Likewise, higher mean glucose levels were found for the women who had a cesarean section than for those who had a vaginal delivery (80.4 mg/dL and 78.0 mg/dL, respectively, p = 0.034). Women who had a cesarean section were also found to have higher mean fasting glucose levels in the third trimester than those who had a normal delivery (80.4 mg/dL and 77.1 mg/dL, respectively, p = 0.005, Table 3).

**Table 3:** **Maternal fasting plasma glucose levels per trimester, birth weight and mode of delivery (Rio de Janeiro, Brazil - 1999 and 2008)**

***Weight at birth (Kg)***	***Fasting plasma glucose in the first trimester (mg/dL)***				***Fasting plasma glucose in the second trimester (mg/dL)***				***Fasting plasma glucose in the third trimester (mg/dL)***			
	**N**	**Mean**	**SD**	**p**	**N**	**Mean**	**SD**	**p**	**N**	**Mean**	**SD**	**p**
Underweight	9	76.1	10.2	0.585	15	78.7	12.3	0.005	8	82.6	14.7	0.545
Normal	286	79.3	10.3		412	78.8	9.9		342	78.7	10.6	
*High (macrosomia)*	15	78	7.6		24	86.2	19.7		21	80	11.6	
*Post-hoc (p)*						86.2 > 78.8 (0.003)						
***Mode of delivery***												
Vaginal	173	78.8	9.4	0.582	244	78.0	9.3	0.039	194	77.1	10.0	0.007
Cesarean	139	79.4	11.0		218	80.4	11.5		193	80.4	10.9	
Forceps	15	76.6	12.6		18	77.9	12.5		15	80.1	11.4	
*Post-hoc (p)*						80.4 > 78.0 (0.034)ª				80.4 > 77.1 (0.005)^b^		

p = *Post-hoc.*

^a^Fasting plasma glucose in the second trimester; ^b^fasting plasma glucose in the third trimester.

Maternal fasting plasma glucose levels were divided into three ranges (<80 mg/dL, 80-90 mg/dL and 90-94 mg/dL), and categorized per trimester of pregnancy, then related to the adverse outcomes under study. This analysis could not be done for fasting plasma glucose in the first trimester because of the limited number of observations of this variable in the different ranges studied. Most of the women evaluated in the first trimester of pregnancy (53%) had fasting glucose levels of <80 mg/dL.

The percentage of the three maternal fasting glucose concentration ranges in the second trimester were: 58.3% <80 mg/dL, 28.5% 80-90 mg/dL, and 13.2% 90-94 mg/dL. The probability by risk of GDM was higher amongst the women in the 90- 94 mg/dL range (OR =7.2; 95% CI =2.33-22.24) than it was for the women with lower levels (see Table 4). No association was found between the plasma glucose ranges studied and the pregnancy complications macrosomia and cesarean delivery for the second trimester of pregnancy (Table 4).

**Table 4:** **Maternal fasting plasma glucose levels in the second and third trimesters, divided into three ranges, and outcomes (Rio de Janeiro, Brazil - 1999 and 2008)**

***Fasting glucose (mg/dL)***	***n (%)***	***GDM n (%) OR (95% CI)***		***Macrosomia n (%) OR (95% CI)***		***Cesarean section n (%) OR (95% CI)***	
***2nd Trimester***							
<80	168 (58.3)	6 (3.6)	1.0	10(3.9)	1.0	110 (40.7)	1.0
80-90	82 (28.5)	4 (4.9)	1.38 (0.38-5.04)	8 (5.7)	1,33 (0.50-3.51)	77 (51.3)	1.36 (0.89-2.08)
90-94	38 (13.2)	8(21.1)	7.2 (2.33-22.24)	6(10.9)	2.03 (0.65-6.33)	31 (51.7)	1.19 (0.66-2.16)
***3rd Trimester***							
<80	140 (62.5)	1 (0.7)	1.0	13(6.0)	1.0	104 (45.2)	1.0
80-90	65 (29)	2 (3.1)	4.4 (0.39-49.56)	5 (4.6)	0.73 (0.25-2.13)	60 (49.6)	1.24 (0.77-1.98)
90-94	19 (8.5)	1(5.3)	7.7 (0.46-128.9)	3 (6.7)	0.77 (0.16-3.62)	29 (58.0)	1.86 (0.95-3.65)

OR: odds ratio; 95% CI: 95% confidence interval.

Adjusted for the following variables: maternal age, parity, pre-pregnancy BMI.

With women diagnosed as having GDM in the second trimester excluded from the analysis of the third trimester.

In the third trimester, 62.5% of the women had fasting plasma glucose levels of <80 mg/dL; 29% fell into the 80-90 mg/dL category, and 8.5% in the 90–94 mg/dL category. No association was found between the plasma glucose ranges studied and the pregnancy complications and outcomes studied (GDM, macrosomia, cesarean delivery) for the third trimester of pregnancy (Table 4).

## Discussion

GDM can have negative effect on maternal and newborn health. Therefore, reducing the risk of perinatal morbidity and mortality from pregnancy complications is a focus of study in modern obstetrics [2]. This study showed that even when second and third trimester fasting glucose is below the established cut-off values for the diagnosis of GDM, it is associated with an increased risk of pregnancy complications as macrosomia, GDM and cesarean section.

In the HAPO study [4], a significant association was found between increasing maternal fasting plasma glucose concentrations in the second trimester, and the increased occurrence of fetal macrosomia, need for surgical delivery, neonatal hypoglycemia, and preeclampsia [4,5]. In the anthropometric evaluation of the pregnant women in the HAPO study, their average pre-pregnancy BMI was 27.7 kg/m^2^, and most of them were overweight at onset of pregnancy. A sub-analysis was carried out on the same cohort [21] to evaluate whether GDM and maternal obesity were associated individually or jointly with adverse fetal outcomes or maternal complications. It was found that women with GDM who were not obese were more at risk of having outcomes such as fetal macrosomia, shoulder dystocia or birth injury, a higher percentage of body fat in the newborns, and a higher concentration of cord-blood serum C-peptide than the obese women without GDM. Our cohort had a higher prevalence of normal weight women, which reinforces the importance of high maternal glucose as a risk factor for adverse pregnancy outcomes.

Alongside pre-pregnancy BMI, we found other differences between the cohorts of the HAPO study [4] and this study. For instance, average maternal age, mean fasting glucose levels at different stages of pregnancy, and prevalence of PIH were all higher in the HAPO study [4].

The higher prevalence of PIH and fetal macrosomia in the HAPO study [4] could be attributed to the higher prevalence of pre-pregnancy overweight in that study than in ours. The association between maternal overweight/obesity and both PIH and macrosomia has been described in other studies [22,23].

In this study, the mean maternal fasting glucose levels were calculated for each trimester of pregnancy. A similar approach was taken in a study of 6129 women from June 2001 to June 2006 [5]. In that study, the authors assessed the association between maternal fasting glucose levels in the first trimester of pregnancy and certain adverse outcomes, including fetal macrosomia, surgical delivery and GDM. They divided fasting glucose into seven ranges: <75 mg/dL, 75-79 mg/dL, 80-84 mg/dL, 85-89 mg/dL, 90-95 mg/dL, 95-99 mg/dL, 100-105 mg/dL. It was found that the higher the maternal fasting glucose level, the greater the likelihood of perinatal complications. The risk of GDM, fetal macrosomia, and delivery by cesarean section was higher for the women whose fasting glucose was 80-84 mg/dL in the first trimester [5].

In this study, we found that fasting glucose levels in the second trimester above 90.5 mg/dL, 86.2 mg/dL and 80.4 mg/dL were potential markers by factors of GMD, macrosomia and cesarean section, respectively.

Another study that has demonstrated an association between maternal glucose below the classic cut-off values for the diagnosis of GDM and adverse pregnancy outcomes involved 14036 women diagnosed as not having GDM, but with one elevated glucose value in the oral tolerance test. The study demonstrated increased rates of cesarean deliveries, fetal macrosomia (weight ≥4000 g), preeclampsia, and admissions to neonatal intensive care units [24] for this group than for the women whose test values were all normal.

An uncontrolled study conducted between 2000 and 2009 evaluated the frequency of adverse pregnancy and neonatal outcomes in women with positive screening (fasting plasma glucose ≥ 90 mg/dL and/or risk factors for GDM) but negative diagnostic test for GDM (glycemic index 3 h after 100 g glucose intake anhydrous under two cutoff points above normal values for the diagnosis of GDM). It reported similar data to the other studies, such as cesarean deliveries in 62.3% of the women studied, 14.2% preterm deliveries, and 19.3% newborns classified as large for gestational age [8].

The plasma glucose ranges associated with the outcomes studied were consistent with those published in a new diagnosis proposed by the IADPSG in 2010, reinforcing the need for further studies into the influence of maternal plasma glucose concentrations and the gestational period in which these are most related to the occurrence of adverse pregnancy outcomes.

The study showed that maternal glucose levels in the second trimester of pregnancy were associated with the risk of developing complications such as GDM. It was demonstrated that fasting glucose testing, an easy, low-cost exam, has great potential for the early detection of women at risk of having more adverse perinatal outcomes, irrespective of the presence of other risk factors, such as obesity and maternal age.

The findings were very important and corroborate those already in the literature on the subject in question. Emphasized the need for earlier onset of possible prenatal care for the screening and diagnosis of GDM for all pregnant women for the prevention of the consequences of this.

The maternal glycemia, even below the classically considered values for the diagnosis of GDM in Brazil, appeared as a short and long-term risk factor for the health of the mother and her son, demonstrating the importance of reducing the cutoff points for the diagnosis of GDM consolidated by IADPSG [9] and the ADA [1], at the earliest assessment of these women in prenatal care. The gestational periods in which maternal blood glucose had greater influence on the appearance of complications were the second and third quarters, indicating the need for care further enhanced in these vulnerable periods, since they are related to intensive development phase and fetal growth.

This study has some limitations that should be considered: the cross-sectional research design does not allow cause-effect inferences to be made between the variables investigated, and the sample size is smaller than in the other studies investigating the topic.

## Conclusion

Even when second and third trimester fasting glucose is below the established cut-off values for the diagnosis of GDM, it is associated with an increased risk of pregnancy complications. Thus, this study with other literature on the subject, point to the need of the earliest possible start of prenatal care for the screening and diagnosis of GDM for all pregnant women including those considered as low risk pregnancy, for the prevention of consequences of this.

## Abbreviations

GDM: Gestational diabetes mellitus
SD: Standard deviation
HAPO: Hyperglycemia and adverse pregnancy outcome
IADPSG: International Association of Diabetes and Pregnancy Study Groups
CI: Confidence interval
ICC: Intraclass correlation coefficient
*k*: Kappa

## References

[1] American Diabetes Association (ADA) (2012). Standards of medical care in diabetes. Diabetes Care.

[2] Diagnostic Criteria and Classification of Hyperglycemia First Detected in Pregnancy. World Health Organization; 2013. Acesso em: http://apps.who.int/iris/bitstream/10665/85975/1/WHO_NMH_MND_13.2_eng.pdf. 24199271

[3] Ferrara A, Hedderson MM, Albright CL, Ehrlich SF, Quesenberry CP, Peng T (2011). Pregnancy and postpartum lifestyle intervention in women with gestational diabetes mellitus reduces diabetes risk factors: a feasibility randomized control trial. Diabetes Care.

[4] HAPO Study Cooperative Research Group (2008). Hyperglycemia and adverse pregnancy outcomes. New Engl J Med.

[5] Riskin-Mashiah S, Younes G, Damti A, Auslender R (2009). First-trimester fasting hyperglycemia and adverse pregnancy outcomes. Diabetes Care.

[6] Schmidt MI, Duncan BB, Reichelt AJ, Branchtein L, Matos MC, Forti AC (2001). Gestational diabetes mellitus diagnosed with a 2-h 75-g oral glucose tolerance test and adverse pregnancy outcomes Brazilian Gestational Diabetes Study Group. Diabetes Care.

[7] Schmidt MI, Matos MC, Reichel AJ, Forti AC, Lima L, Duncan BB (2000). Prevalence of gestational diabetes mellitus – do the new WHO criteria make a difference?. Diabet Med.

[8] Rehder PM, Pereira BG, Silva JLP (2011). Resultados gestacionais e neonatais em mulheres com rastreamento positivo para diabetes mellitus e teste oral de tolerância à glicose - 100 g normal. Rev Bras Ginecol Obstet.

[9] International Association of Diabetes and Pregnancy Study Groups Consensus Panel (IADPSG) (2010). International Association of Diabetes and Pregnancy Study Groups Recommendations on the diagnosis and classification of hiperglycemia in pregnancy. Diabetes Care.

[10] Clausen T, Burski TK, Oyen N, Godang K, Bollerslev J, Henriksen T (2005). Maternal anthropometric and metabolic factors in the first half of pregnancy and risk of neonatal macrosomia in term pregnancies. A prospective study. Eur J Endocrinol.

[11] Mahony R, Foley M, Mcauliffe L, O’Herlihy C (2007). Maternal weight characteristics influence recurrence of fetal macrosomia in women with normal glucose tolerance. Aust N Z J Obstet Gynecol.

[12] Nicoll AE, Black C, Powls A, Mackenzie F (2004). An audit of neonatal respiratory morbidity following elective caesarean section at term. Scott Med J.

[13] Weiderpass E, Barros FC, Victora CG, Tomasi E, Halpern R (1998). Incidência e duração da amamentação conforme o tipo de parto: estudo longitudinal no Sul do Brasil. Rev Saúd Públ.

[14] Rothman KJ, Greenland S, Lash TL (2011). Tipos de estudos epidemiológicos. Epidemiologia Moderna.

[15] American Diabetes Association (ADA) (2000). Gestational diabetes mellitus [Position Statement]. Diabetes Care.

[16] American Diabetes Association (ADA) (2003). Gestational diabetes mellitus [Position Statement]. Diabetes Care.

[17] Kathleen M, Catalano PM, Yaktine AL (2009). Committee to reexamine IOM pregnancy weight guidelines; Institute of Medicine; National Research Council.

[18] Brasil. Ministério da Saúde. Secretaria de Atenção à Saúde. Departamento de Ações Programáticas Estratégicas (2005). Pré-natal e Puerpério: atenção qualificada e humanizada – manual técnico/Ministério da Saúde, Secretaria de Atenção à Saúde, Departamento de Ações Programáticas Estratégicas.

[19] Saunders C, Padilha PC, Chagas CB, Silva CL, Accioly E, Ramalho A (2009). Consistência das informações de um estudo sobre o impacto da assistência nutricional no pré-natal. Rev Paul Pediatr.

[20] Landis JR, Koch GG (1977). The measurement of observer agreement for categorical data. Biom.

[21] Hapo Study Cooperative Research Group (2012). Hyperglycemia and adverse pregnancy outcomes. Associations of GDM and obesity with pregnancy outcomes. Diabetes Care.

[22] Nucci LB, Schmidt MI, Duncan BB, Fuchs SC, Fleck ET, Britto MMS (2001). Nutritional status of pregnant women: prevalence and associated pregnancy outcomes. Rev Saúd Públ.

[23] Seabra G, Padilha PC, Queiroz JA, Saunders C (2011). Sobrepeso e obesidade pré-gestacionais: prevalência e desfechos associados à gestação. Rev Bras Ginecol Obstet.

[24] Mclaughlin GB, Cheng YW, Caughey AB (2006). Women with one elevated 3-hour glucose tolerance test value: are they at risk for adverse perinatal outcomes?. Am J Obst Gynecol.

